# Case Report: Complete recovery of a patient with occult septic shock following supranormal vasopressor and inotrope therapy informed by the novel ‘pressure field method’ for managing hemodynamics

**DOI:** 10.3389/fmed.2025.1552050

**Published:** 2025-05-09

**Authors:** Stephen F. Woodford, Ruth C. Marshall

**Affiliations:** ^1^Department of Critical Care, University of Melbourne, Parkville, VIC, Australia; ^2^Department of Anesthesia, Austin Health, Heidelberg, VIC, Australia; ^3^Department of Anesthesia and Intensive Care, Brisbane Waters Private Hospital, Woy Woy, NSW, Australia

**Keywords:** pressure field, septic shock, elastance, perfusion, personalized hemodynamics, norepinephrine

## Abstract

Complete recovery of a patient with occult septic shock and left ventricular ejection fraction of 10% was achieved following management informed by continuous measurement and visualization of the patient’s ‘pressure field’. The ‘pressure field’ visualizes mean perfusion pressure as the product of stroke volume and a beat-to-beat measure of vascular tone, termed systemic elastance. The pressure field guided the titration of inotropes and vasopressors at high doses, including norepinephrine equivalents >2.5 μg/kg/min, to restore the patient’s estimated pre-morbid pressure field values. Urine output was maintained throughout with no ileus. We hypothesize that pressure field management assists in individualizing care for patients with septic shock and improves outcomes.

## 1 Introduction

Septic shock has an in-hospital mortality rate of 30%–40% (1, 2), a 5-year mortality rate of 80% (3), and morbidity among survivors. Patients with sepsis-induced myocardial dysfunction have even poorer outcomes with in-hospital mortality rates of 50%–80% (4), and management may be further complicated by the fact that the diagnosis (cardiogenic or septic shock) is uncertain at presentation. We report a patient with occult septic shock due to *Klebsiella aerogenes* pneumonia, initially presenting with fatigue, hypotension, and rapid atrial fibrillation with a history of viral cardiomyopathy. The patient was managed using the novel ‘pressure field method’ for managing hemodynamics, made a complete recovery, and remains well over 5 years later. Brief summaries of four additional septic shock cases managed with the pressure field method are provided as comparators.

### 1.1 Pressure field method

**Figure 1:**
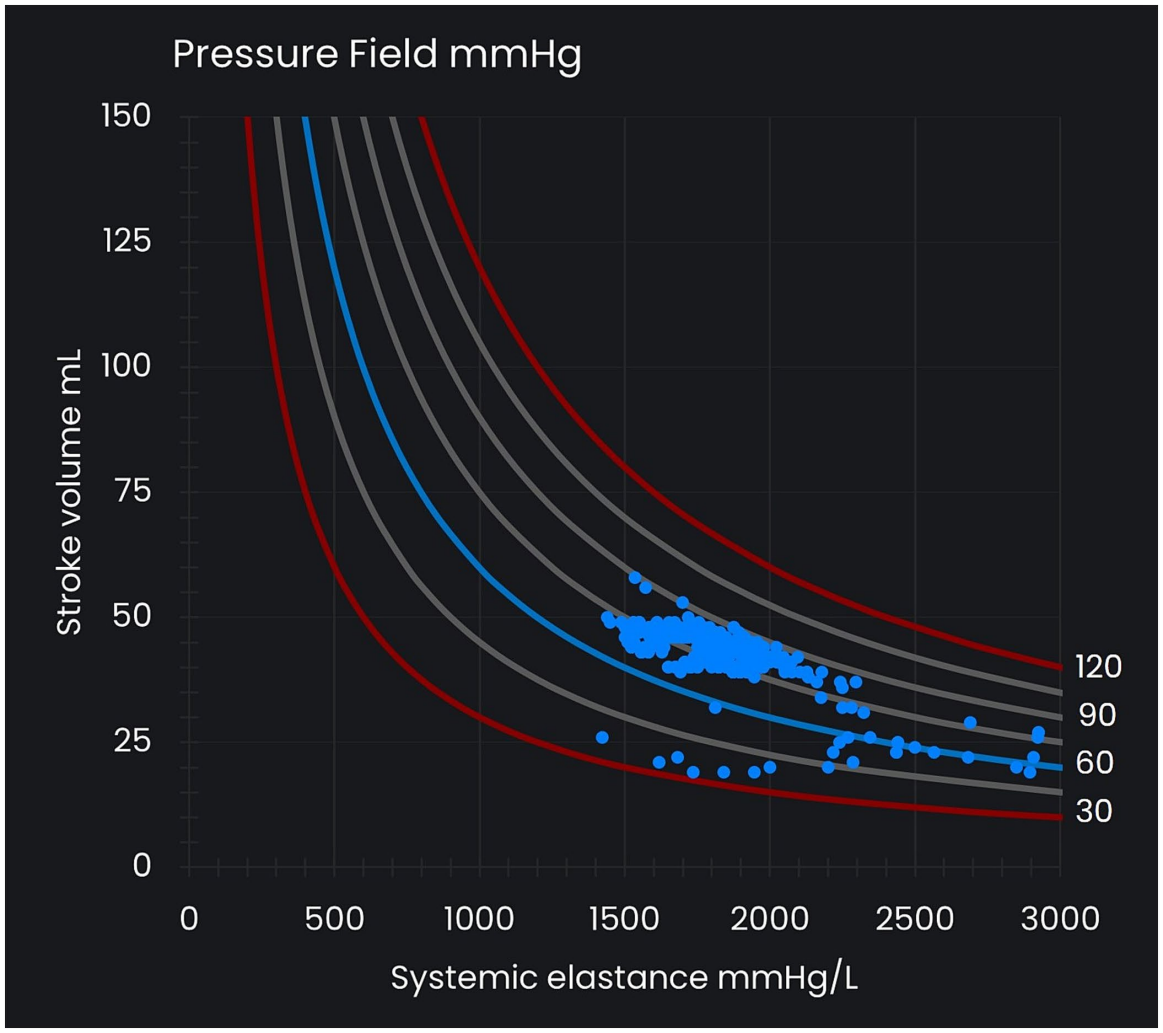
Pressure field visualization. The x-axis represents systemic elastance (E_sys_) as a measure of vascular tone, and the y-axis represents stroke volume (SV), which varies with preload and contractility. The curved lines represent reference lines for mean perfusion pressure (MPP) at intervals of 15 mmHg and between 30 mmHg and 120 mmHg. Each MPP value (that is, [MAP – CVP] value) is consistent with many different SV and E_sys_ values. Each dot in the pressure field visualization represents a time/data point.

The ‘pressure field visualization’ resolves blood pressure into its cardiac and vascular components and displays this ventricular–vascular relationship to support clinical decision-making (see Figure 1). The visualization is based on the ‘pressure field equation,’ which expresses blood pressure as the result of the *beat-to-beat* contributions of the heart and vasculature such that


(1) $$MPP=\left[\text{MAP}-CVP\right]=SV\times E_{sys},$$

where MPP is mean perfusion pressure, MAP is mean arterial pressure, CVP is central venous pressure, SV is stroke volume, and E_sys_ is systemic elastance (5–9). This finding is similar to the more familiar Starling and Guyton equation ([MAP – CVP] = CO × (systemic vascular resistance) SVR), but the time-averaging has been removed from all elements, leaving only the instant values (see equation derivation in Supplementary material 1). E_sys_ is a more sensitive measure of vascular tone than SVR (10), especially during shock states when heart rate is commonly high and variable (5, 6, 9). Blood pressure and SV values from a minimally invasive cardiac output monitor are used to calculate and display the pressure field visualization.

Ongoing visualization of a patient’s pressure field informs the choice and titration of hemodynamic therapy with the goal of restoring a patient’s estimated pre-morbid pressure field values (‘pressure field zone’). Changes in the x-axis of the pressure field visualization (changes in E_sys_) represent changes in vascular tone and are managed with vasopressors titrated against the physiological response. Changes in the y-axis (SV) are the result of changes in preload and contractility and are managed with fluid and inotropes.

## 2 Case description

### 2.1 Presentation of patient

A 54-year-old man was diagnosed with viral cardiomyopathy (left ventricular ejection fraction 10%–15%) by his cardiologist, but he discontinued medications several months later after being lost to follow-up. Three years after the diagnosis of viral cardiomyopathy, he (at 57 years of age and 90 kg) presented to his family physician with a rapid pulse following 3 days of increasing fatigue. His family physician recommended ambulance transfer to our hospital; he initially refused but presented to our hospital several hours later that same day. On arrival, he was hypotensive (BP 70/50) with rapid atrial fibrillation (150–180 bpm), cool peripheries, and confusion. A flecainide infusion (150 mg) and intravenous metoprolol (5 mg) were administered, restoring sinus rhythm. The patient progressed to pulmonary edema, became diaphoretic and confused, and was transferred to the intensive care unit (ICU).

### 2.2 Initial ICU crisis response

**Figure 2:**
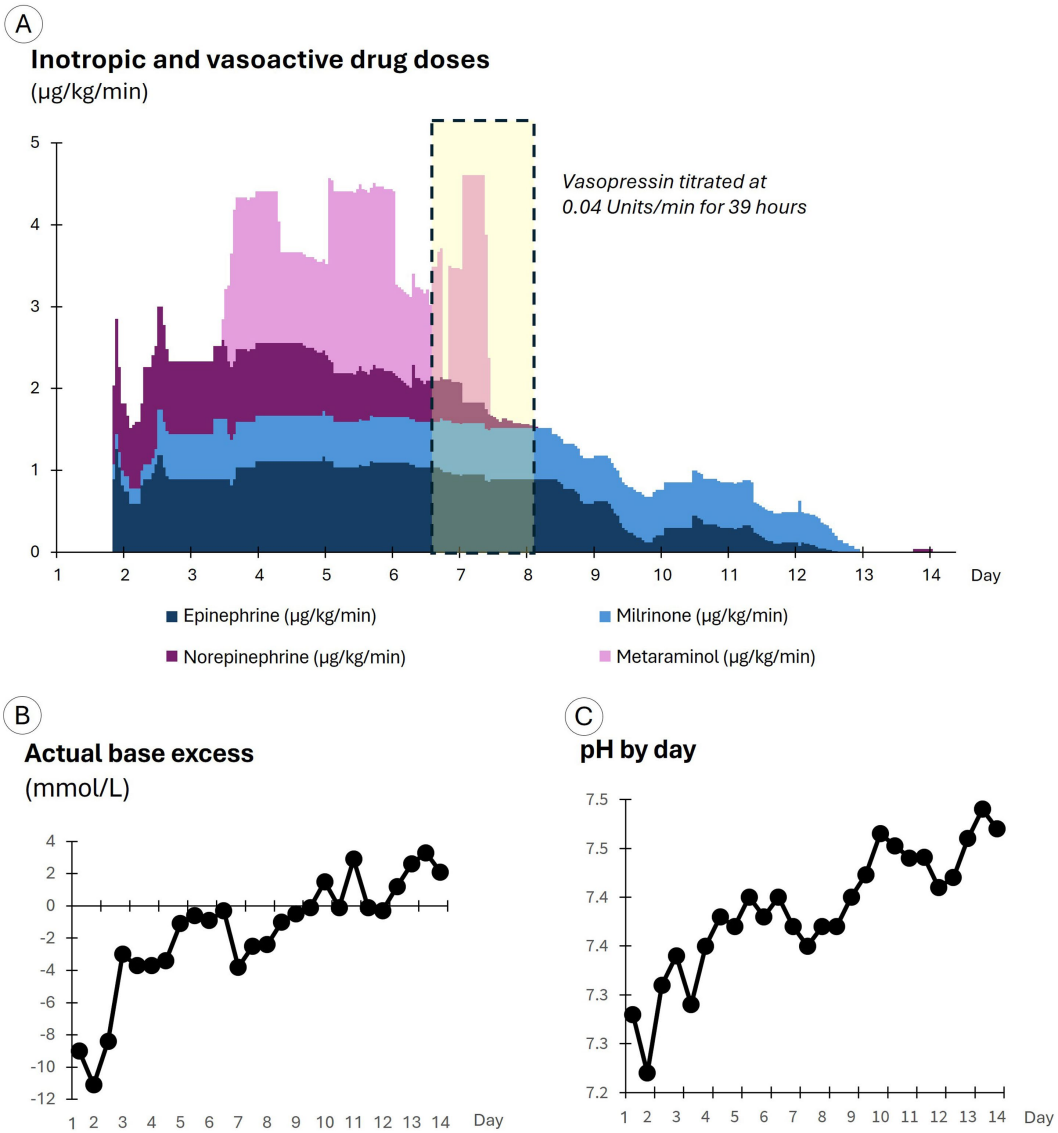
Drug doses and arterial blood gas results by day of care. **(A)** Displays inotropic and vasoactive drug doses. **(B)** Displays the actual base excess results. **(C)** Displays pH results.

A central line was inserted, and epinephrine was initiated and titrated up to 1.1 μg/kg/min to support blood pressure (see Figure 2). Minimally invasive cardiac output monitoring was then urgently commenced using the Edwards Lifesciences (CA, United States) EV1000 monitoring platform with a radial arterial line connected via a FloTrac transducer (v3.0) and transduced central venous pressure (CVP). The patient’s MAP was 80 mmHg, CVP 21 mmHg, and SV 45 mL/beat. The patient was intubated, and transesophageal echocardiography demonstrated profoundly depressed biventricular function with a left ventricular ejection fraction of 10%. Arterial blood gases (ABGs) showed pH 7.28, actual base excess (ABE) -9.0 mmol/L, and P_a_O_2_/F_i_O_2_ ratio 103 (see Figure 2). A full blood count indicated a raised white cell count (34 × 10^9^/L), but there were no focal signs of bacterial sepsis.

The cause of the shock was unclear. A dilated cardiomyopathy with acute decompensation, exacerbated by flecainide and metoprolol, appeared most likely. The elevated white cell count was initially interpreted as likely to be a stress response rather than the result of infection. Mechanical circulatory support was unavailable. Septic shock could not be definitively excluded.

### 2.3 Ongoing ICU management

**Figure 3:**
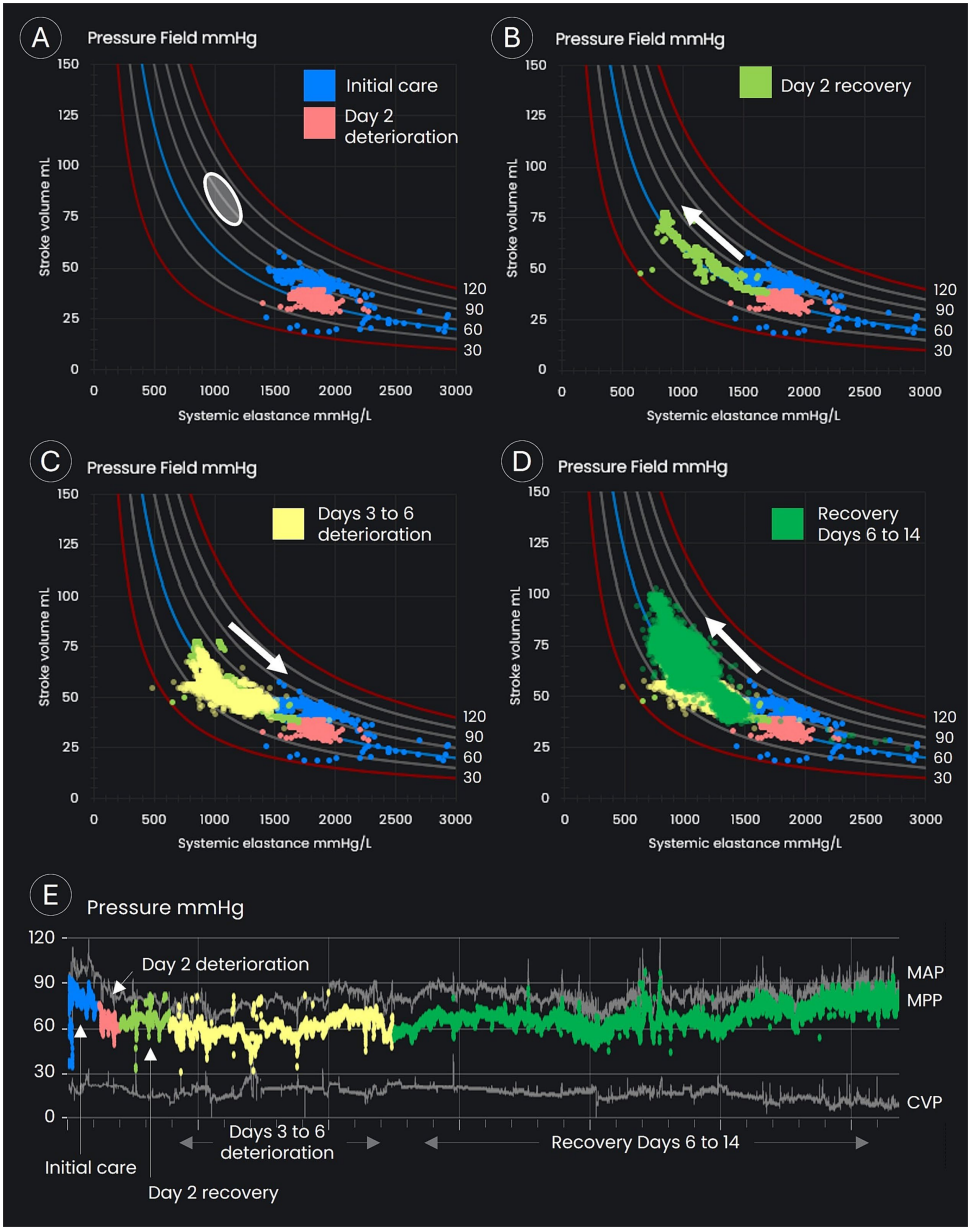
Pressure field visualization throughout the period of intensive care. **(A–D)** Display time/data points in the pressure field visualization. **(A)** Displays the estimated pre-morbid pressure field values (the white oval), the data points on day 1 and early on day 2 (the blue dots), and the period of deterioration on day 2 (the orange dots). **(B)** Displays the recovery in stroke volume on day 2 following up-titration of inotropes and vasopressors. **(C)** Displays the slow deterioration over days 3 to 6, with a decline in stroke volume and an increase in systemic elastance. **(D)** Displays the period of recovery back toward the patient’s estimated pre-morbid pressure field values from day 6 onward. **(E)** Displays blood pressures for the complete monitoring period.

Following the initial crisis response, the patient’s pressure field was visualized in Excel from files downloaded from the Edwards Lifesciences hemodynamic monitoring platform. The pressure field demonstrated that the patient’s MPP had dropped to 55 mmHg shortly following ICU admission with an SV of 25–45 mL/beat and E_sys_ of 1,200–2,200 mmHg/L (see the blue dots in Figure 3A). In contrast, the patient’s pre-morbid pressure field zone was estimated to be an MPP of 80–100 mmHg and an SV of 70–90 mL/beat (see the white circle in Figure 3A). The pressure field visualized hemodynamic instability with profound myocardial depression and vasoconstriction.

The goal was to achieve an MPP of 80 mmHg with an increased SV, that is, to move the patient’s pressure field toward the estimated pre-morbid zone.

To increase SV, epinephrine was titrated to 0.9 µg/kg/min and milrinone commenced at 0.2 µg/kg/min; and norepinephrine was added and titrated up to 1.0 µg/kg/min to support the heart and vasculature (see Figure 2). In response, blood pressure improved with a small increase in SV. An amiodarone infusion was commenced to prevent the recurrence of atrial fibrillation. Cefazolin (1 g) was administered intravenously because sepsis could not be excluded, and sodium bicarbonate (150 mmol in four doses) was given intravenously to improve acidemia.

On day 2, MPP fell below 60 mmHg and SV to 29 mL/beat (see the orange dots in Figure 3A), and in response, drug doses were further titrated up, with the epinephrine dose reaching 1.3 μg/kg/min, milrinone 0.6 μg/kg/min, and norepinephrine 1.2 μg/kg/min (see Figure 2). The doses of epinephrine and norepinephrine were titrated down to 0.9 μg/kg/min when SV reached 40 mL/beat. SV continued to improve, eventually doubling to 78 mL/beat, although blood pressure increased only marginally and stabilized at an MPP of 55–65 mmHg with a MAP of 75–85 mmHg (see the green dots in Figure 3B). Blood cultures grew Gram-negative rod-shaped bacteria. Antibiotic cover was broadened to include erythromycin (1 g daily) and metronidazole (500 mg three times per day). The Gram-negative rod was later identified as *Klebsiella aerogenes*. Severe cardiomyopathy with overt heart failure, possibly precipitated by infection from an unknown source, still appeared the most likely cause of shock.

Over days 3 to 6, blood pressure was maintained, but SV gradually declined from its peak of 78 mL/beat (see the yellow dots in Figure 3C), despite an average epinephrine dose of 1 μg/kg/min, an average norepinephrine dose of 0.9 μg/kg/min, and the addition of metaraminol on day 3 at an average dose of 1.6 μg/kg/min, with dosage adjustments (see Figure 2) assessed by reviewing the impact on the pressure field. A levosimendan infusion was administered on days 3 and 4 at 0.1 μg/kg/min. Urine output was maintained throughout, and the initial acidemia was resolved by day 5 (see Figure 2).

On day 5, the positive fluid balance reached 15 L, primarily due to the diluents used with infused drugs. Urine output remained unresponsive to diuretics; therefore, although the acidemia had already resolved, continuous renal replacement therapy (CRRT) was initiated to manage fluid overload (see Supplementary Figure S1 for fluid balances). Late that evening, right upper lobe consolidation became apparent (possibly due to the gradual resolution of pulmonary edema), and sepsis appeared increasingly likely as the cause of shock. A single dose of ceftriaxone (2 g) was administered, and erythromycin and metronidazole were continued.

On day 6, MPP fell to 55 mmHg (see the yellow dots in Figure 3C) due to a slow deterioration in SV (a fall to 44 mL/beat), offset partially by an increase in vascular tone (increased E_sys_). Despite severe ventricular dysfunction, vasopressin was commenced at 0.04 units/min. Hemodynamic improvements occurred soon after. Blood pressure and SV increased, while E_sys_ fell overnight and continued to improve the following day (see the green dots in Figure 3D). Metaraminol was discontinued by midday, and norepinephrine was discontinued by the evening of day 7. Vasopressin was ceased early on day 8, at which point MPP had increased to 68 mmHg, MAP to 84 mmHg, and SV to 57 mL/beat.

Epinephrine and milrinone were weaned and ceased over days 8 to 13 as hemodynamics continued to improve. On day 12, with antibiotic sensitivities available, erythromycin and metronidazole were discontinued, and meropenem (1 g three times per day) was commenced, although the patient had largely recovered.

By the morning of day 14, with no vasopressor support, MPP was 77 mmHg, MAP 87 mmHg, and SV 65 mL/beat, with these values approaching the patient’s estimated pre-morbid pressure field values (see the green dots in Figure 3D). The SV recovery suggested that sepsis was the sole cause of biventricular dysfunction.

Hemodynamic monitoring and CRRT were ceased, and the patient was transferred to a lower acuity ward. Ventilatory support was weaned over 10 days before rehabilitation and discharge home. The patient returned to work and remains well more than 5 years later.

Transesophageal echocardiography studies were performed throughout the first week of care. These confirmed profound myocardial dysfunction and subsequent recovery but were of limited use in titrating inotropes and vasopressors.

A care timeline is shown in Supplementary Figure S2 and a video summary in Supplementary Video S1.

## 3 Additional case summaries

A total of 15 additional patients with septic shock have been managed using the pressure field method, and four patients (each with an estimated weight of 90 kg) had similar estimated pre-morbid pressure field values to the patient described in the case.

**Figure 4:**
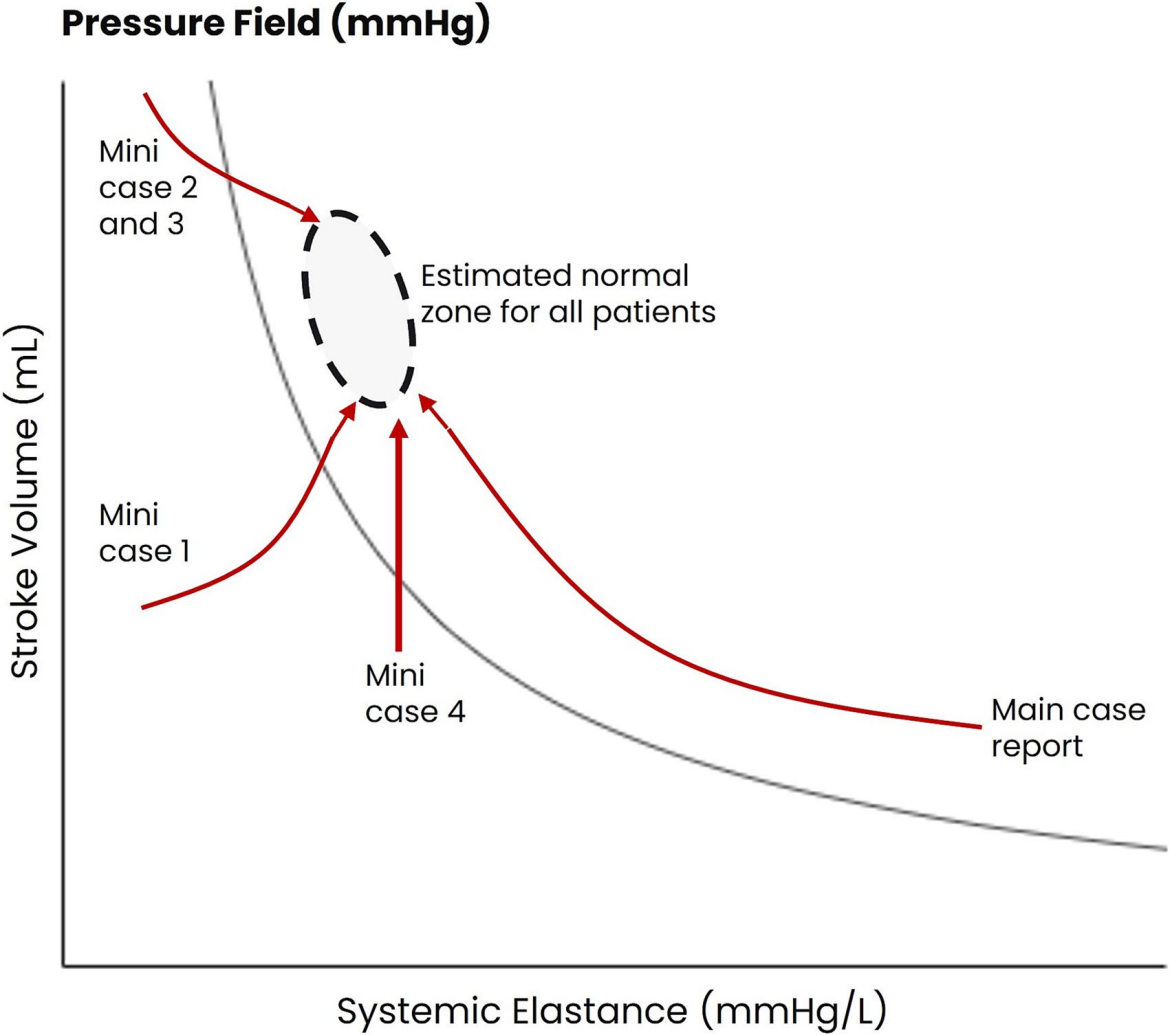
Patterns of hemodynamic movement in patients with septic shock being managed with the pressure field method. The estimated pre-morbid pressure values for four patients are represented by the black oval. The changes in hemodynamic function for the four patients during the pressure field monitoring period are represented by the red arrows.

The patterns of hemodynamic change for these four patients are outlined in Figure 4, with brief case summaries in Supplementary Table S1. These cases illustrate the heterogeneity of hemodynamic insults associated with sepsis: one patient was hypodynamic and vasodilated, two were hyperdynamic and vasodilated, and one was hypodynamic with ‘normal’ vascular tone. These cases also illustrate the tailoring of therapy using the pressure field method: although norepinephrine was administered in each case, the maximum dose per patient varied between 15 and 80 μg/min.

## 4 Discussion

### 4.1 Summary of cases

The primary patient exhibited severe myocardial dysfunction and vasoconstriction. Hemodynamic management was guided by mapping ventricular–vascular interaction using the ‘pressure field visualization’ and targeting the patient’s estimated pre-morbid ‘pressure field zone.’ The patient made a full recovery despite a delayed diagnosis of sepsis, inadequate broad-spectrum antibiotic therapy in the early stages of management, and delay in the commencement of tailored antibiotics. He was fed enterally and experienced no ileus, and urine output was maintained throughout admission despite prolonged administration of epinephrine up to 1.3 μg/kg/min (with an average dose of 1 μg/kg/min over 7 days), norepinephrine (up to 1.4 μg/kg/min, with an average dose of 0.9 μg/kg/min over 4 days), and high doses of metaraminol (an average dose of 1.7 μg/kg/min for 4 days). CRRT was initiated to manage fluid balance but only commenced after acidemia had resolved.

The additional case summaries highlight the heterogeneity of sepsis, even among patients with similar estimated pre-morbid hemodynamic function, and the tailoring of therapy in response.

### 4.2 The pressure field as a method for managing hemodynamics

Challenges in managing shock relate to the heterogeneity of patients’ pre-morbid hemodynamics, the heterogeneous impact of acute disease on these hemodynamics, and the heterogeneity of patient responses to therapy. We propose the ‘pressure field method’ as a framework for addressing this heterogeneity and facilitating individualized management irrespective of the underlying cause of shock. The method is an alternative to population-based disease-specific protocols such as the Surviving Sepsis Campaign guidelines (11), to which strict adherence in this case would have resulted in early fluid loading of a failing heart and acceptance of an MPP of 45 mmHg (that is, a MAP of 65 mmHg with a CVP of 20 mmHg). The method also supports the management of undifferentiated shock, for which there is no broadly accepted guideline.

The pressure field method involves understanding pressure as the product of SV and a pulsatile measure of ventricular afterload termed E_sys_ (see Equation 1, with the equation derivation in Supplementary material 1). Elastance has been argued to be a better measure of ventricular afterload than SVR, as demonstrated by Sunagawa (10, 12) using Otto Frank’s pressure-volume loops (13), and its measurement in the intact circulation is validated (14–16). In the traditional Starling and Guyton pressure equation, CO represents the load transferred by the heart to create blood pressure, and SVR is the resistive force. Clinicians recognize that CO is a composite measure, that can be broken down into SV and heart rate. Equally critically, SVR can be broken down such that


(2) $$SVR=E_{sys}/HR,$$

where SVR = systemic vascular resistance, E_sys_ = systemic elastance, and HR = heart rate. In shock states, both elastance and heart rate commonly increase, so SVR may appear stable (implying no change) or even decrease (implying vasodilation) when there is significant constriction (5, 6, 9) of the arterioles (the main site of resistance in the circulation (17, 18)); see Equation 2. This pattern was observed in the primary patient reported in this study on day 1 during rapid atrial fibrillation. We hypothesize that expressing perfusion pressure in terms of its ventricular and vascular components, SV and E_sys_ (rather than CO and SVR), provides clinicians with more granular information, particularly in relation to vascular tone, and enables earlier, more targeted diagnosis and treatment.

The second element of pressure field management involves identifying and managing personalized hemodynamic zones defined in terms of MPP, SV, and E_sys_. Explicitly estimating a patient’s pre-morbid pressure field values usefully informs management. We intend to publish further guidance; however, a useful rule of thumb for the normal adult population is an SV of 1.1 mL/kg/beat and an MPP of 80–100 mmHg (9). For the primary patient reported, given the history of viral cardiomyopathy, the target SV was revised downward.

The third element of pressure field management involves visualizing a patient’s pressure field in a graph with SV on the y-axis and E_sys_ on the x-axis (see Figure 1). This graph provides an instantaneous view of how SV and E_sys_ relate to a particular MPP, and sequential plotting of this interaction enables the detection of small directional changes in the beat-to-beat ventricular and vascular contributions to blood pressure. This image provides an *integrated* picture of patient physiology *over time* and thus provides a simple and intuitive means of understanding disease progression and changes in response to treatment.

The pressure field substantially addresses the need to differentiate between preload, afterload, and contractility: changes in the x-axis (changes in E_sys_) represent changes in vascular tone and are managed with vasopressors and vasodilators, with the dose being titrated against a physiological response. Changes in the y-axis (SV) are the result of changes in preload and contractility and are managed with fluids and inotropes.

In managing patients with septic shock, there are typically many steps forward and backward, requiring many therapy adjustments. The pressure field provides a visual indication of what works and what does not, as well as visual evidence of the progression or recovery of vasoplegia and myocardial dysfunction.

### 4.3 High-dose inotropic and vasopressor therapy

In the primary case reported, application of pressure field management resulted in the administration of sustained high doses of inotropes and vasopressors. High-dose inotropes and vasopressors have been associated with high mortality (19, 20). Brown retrospectively studied mortality in critical care patients receiving “high dose vasopressor therapy” (defined as >1 μg/kg/min for more than 10 min of norepinephrine equivalents; n = 443) and found an overall mortality of 83% at 90 days. The survivor and non-survivor groups had median maximum doses of 1.4 μg/kg/min and 1.8 μg/kg/min, respectively, administered for median durations of 2 and 3 h (21). This study was not able to control for disease impacts versus drug toxicity. However, in the primary patient reported here, the maximum norepinephrine-equivalent dose was 2.7 μg/kg/min, and doses >1.4 μg/kg/min were administered for more than 5 days (excluding metaraminol, aligned with Brown’s study). In three of the four additional case summaries, increasing perfusion pressure with norepinephrine was followed by improved renal function, and renal replacement therapy was not required.

We hypothesize that the pressure field visualization combined with personalized hemodynamic zones enables safe and effective use of inotropes and vasopressors, including at high doses. Response to drugs varies by individual patient, and the pressure field visualization enables individual responses to be understood and doses to be precisely titrated.

### 4.4 Future directions

For the primary patient reported, we visualized the pressure field by manually plotting hemodynamic data. We subsequently developed real-time pressure field monitoring software. This has been used to apparent good effect in managing septic patients (see Supplementary material 3) and patients undergoing major surgery (5–9). A prospective trial of pressure field management is being conducted in major abdominal surgery (ACTRN12624000713594p) (22). Prospective studies in patients with suspected or confirmed septic shock are warranted.

### 4.5 Strengths and limitations

Strengths include detailed data from the primary patient reports and the outline of a novel generalizable framework for managing hemodynamics in shock.

FloTrac technology was used to estimate SV, and a meta-analysis has concluded that its accuracy and trending capabilities are sufficient under normo-dynamic or hypodynamic conditions (23). There is some evidence that rapid vascular tone changes induced by vasopressors may make SV estimations less reliable (24); however, changes in sepsis typically occur over hours rather than minutes.

The results are likely influenced by case-specific characteristics, and prospective studies are required to compare the efficacy of the pressure field method with standard treatments.

## 5 Conclusion

We propose the pressure field as a method for managing the heterogeneity of septic shock. This novel method explicitly aims to estimate and restore a patient’s normal hemodynamics—defined by MPP, SV, and E_sys_—to tailor fluid, vasopressor, and inotropic therapy accordingly. This approach is generalizable to the management of other shock types. Patients experiencing shock are individuals, with different pre-morbid hemodynamics, different acute insults, and different responses to therapies. Rather than a protocol for standardizing care, the pressure field is a method—a system—for individualizing care. We hypothesize that significant improvements in patient outcomes are feasible through the adoption of the pressure field method, which addresses the clinical need to match treatment to the disease. Therefore, a prospective observational study of patients with septic shock is warranted.

## Data Availability

The data analyzed in this study is subject to the following licenses/restrictions: data sets are available on request. The raw data supporting this case report will be made available by the authors, without undue reservation. Requests to access these datasets should be directed to steve.woodford@austin.org.au.
